# Resilient Resources in Youth Athletes and Their Relationship with Anxiety in Different Team Sports

**DOI:** 10.3390/ijerph17155569

**Published:** 2020-08-01

**Authors:** Juan González-Hernández, Marcial Gomariz-Gea, Alfonso Valero-Valenzuela, Manuel Gómez-López

**Affiliations:** 1 Department of Personality, Evaluation and Psychological Treatment, University of Granada, 18071 Granada, Spain; jgonzalez@ugr.es; 2 Department of Physical Activity and Sport, Faculty of Sport Sciences, University of Murcia, Santiago de la Ribera, 30720 Murcia, Spain; marcialrbb@gmail.com (M.G.-G.); avalero@um.es (A.V.-V.); 3 Campus of International Excellence “Mare Nostrum”, University of Murcia, 30720 Murcia, Spain

**Keywords:** handball, basketball, resilience, self-confidence, acceptance, competence

## Abstract

The objective of this study is to show the links and differences in the expressions of competitive anxiety in the face of the existence of resilient resources in young athletes, according to sporting (years of experience) and personal (gender) characteristics. To meet these aims, the participants answered the Resilience Scale (RS-14) and the Competitive State Anxiety Inventory-2R (CSAI-2R). The sample consisted of 241 adolescent handball and basketball players between 14 and 17 years old. Different analyses were performed, including a differential and multivariate descriptive, a correlation, and a multiple regression. The results showed that anxiety was negatively related to resilience in its acceptance dimension. It was shown that girls showed higher levels of somatic anxiety, while boys showed higher levels of acceptance. Statistically significant differences were found in the resources for acceptance in favor of boys, while there were significantly different indicators in somatic anxiety and self-confidence in favor of girls. The sports experience was positively related to resilience and negatively to anxiety. Although the existence of indicators of cognitive anxiety (e.g., recurrent thoughts or rhyming), coaches and athletes need to understand that they are also indicators of a necessary activation for psychological functioning. Channeling such a process through psychological training of different skills will enhance the capacities for self-confidence.

## 1. Introduction

The ability to obtain an optimal and stable psychological state during competition is of utmost importance to coaches and athletes [1,2,3]. Physical, social, cognitive, and emotional factors factor into such functioning, as these resources restore balance when a young athlete is in the face of adversity [4,5,6]. In this sense, the study of anxiety has been one of the most prolific lines of research, due to the emotional responses it provokes and that can affect athletes’ performance [7].

Anxiety, which is considered a psychological response produced as a consequence of the differences between an athlete’s response capacity and the demands of the environment, accompanied by a high degree of psycho-physiological activation [8], shows functional conditions that facilitate adaptation (e.g., helps to prepare for action) but also other dysfunctions that weaken or hinder behavior (e.g., nervousness, impulsiveness or rumination) [9].

Among the different classifications that exist around this psychological response and the instruments used in research to measure it, we must highlight somatic anxiety (physiological activation that a person perceives when faced with a stressful situation) and cognitive anxiety (negative expectations and concerns about oneself and the current situation and its possible consequences) [10].

In sports situations, somatic anxiety often occurs in competitions perceived as significant where there are uncontrollable elements and under conditions of social evaluation that lead to stress and a hormonal response, such as increased levels of cortisol in saliva, or physiological aspects, such as increased heart rate or muscle tension [11,12]. To understand the relationship between anxiety and sports performance, Martens, Vealey and Burton [13] established the multidimensional theory, in which they proposed that there is a negative relationship between cognitive anxiety and performance on the one hand, and a curvilinear relationship between somatic anxiety and sports performance on the other.

Anxiety is also related to sociodemographic variables such as age and gender in relation with sport contexts, due to it being multifactorial. Different studies carried out in the sports context have shown that the older the athlete, the greater the experience and competitive level in practicing a sport, and the lower their levels of anxiety, because more experienced athletes have greater and better control over their emotions [14,15], greater technical-tactical mastery, and greater experience in stressful and adverse competitive situations that favor self-confidence [16] and cognitive abilities [17]. With regard to gender, after an extensive review of the literature, no studies were found that show statistically significant differences, although it is true that some studies have shown that women have higher levels of anxiety and less self-confidence than men, and that the production of anxiety in men and women can come from different routes. In men it is be produced by the poor management of a situation, and in women by their level of motivation [12,18,19]. Likewise, with regard to the type of sport practiced, the results of the literature review indicate that athletes who practice team sports in which there is physical contact show higher levels of anxiety than athletes who practice individual sports [17,20].

In a confluent direction with anxiety, resilience protects young athletes from stressful situations or threatening scenarios, giving athletes the means to avoid them [21]. Among the many definitions of resilience, Deen, Truner and Wong [22] explained it as a set of cognitive aspects and behavioral responses to acute or chronic adversity. Luthar, Cichetti and Becker [23] defined it as a dynamic process of positive adaptations within a context of adversity. Fletcher and Sarkar [24] argued that resilience is based on overcoming adversity and on positive adaptation. On the other hand, Wu et al. [25] highlighted effective efforts to environmental challenges and ultimate resistance to the unhealthy effects of stress.

Studied in sports contexts, resilience, like anxiety, is a variable that is dependent on different factors, such as emotions, supports, experiences, strategies, motivation, and self-concept [5,26,27], all of which facilitate optimal sports performance. This concept is really important in youth, with important implications in cognitive, physical, and social development that occurs in the adolescence [28], and its influence on growth on the personal level and in sporting abilities [5,29].

As for the relationship between resilience and the sociodemographic variables of age and gender, it has not been possible to establish firm conclusions since resilience has been little studied in the context of sports [30]. However, some studies have shown that athletes are more resilient, which is probably due to their greater experience in the face of adversity both in professionals [15] and youth athletes [28,31,32]. With respect to their relationship with gender, there are contradictions, as there are studies that have not shown significant differences in the levels of resilience between men and women, and others that point to higher levels of resilience in men caused by a better perception of personal competence [33].

With regard to the relationship between resilience and sports variables such as sporting experience or the sports modality practiced, the results of the review carried out on the first of the variables show contradictory results, since there are studies that, although they have not obtained statistically significant outcomes, have found that professional athletes with higher levels of competence are more resilient than amateur athletes [34,35]; on the other hand, there are works that have reflected that athletes with less sporting experience, like youth athletes, had more resilience capacity [32]. As for the sport practiced, different studies have shown that athletes who practice individual sports have higher levels of resilience [28,36].

Self-confidence is understood as conviction (based on what has been learned and lived before) about the ability to perform a certain task successfully. It is evident that young people have less sporting experience and a greater probability of not being confident in their own skills and resources (e.g., several consecutive errors or failures would affect the perception of self-confidence), which would lead to living with greater perceptions of anxiety. However, different aspects could generate their improvement by looking for self-reflection before the tasks [37], reducing the dramatism for the mistake [38] or generating support bonds with colleagues [39] and rational expression of emotions [40].

Moreover, regarding the relationship between anxiety and resilience in sports contexts, it should be noted that based on the literature review, there is little scientific evidence on this issue in adolescent samples and outside the clinical setting. As an example, Martin-Krumm, Sarrazin, Peterson and Famose [41], in a study conducted in France with adolescent children, demonstrated the relationship between resilience and anxiety, since the results reflected that children with a higher level of resilience controlled their anxiety levels better when performing an ability. Likewise, Ortín-Montero, De la Vega and Gonsálvez-Botella [42], in their study about optimism, anxiety, and self-confidence in handball players, showed that the most resilient players faced the competition with greater confidence and with lower levels of anxiety.

Therefore, and on the basis of what has been reviewed so far, the objectives of the study are as follows: (a) to describe the levels of resilience and competitive anxiety, (b) to analyze the differences in terms of gender and years of sports experience, (c) to study the relationship between resiliency resources and competitive anxiety, and (d) to examine the variables that predict both resiliency resources and self-confidence in players.

## 2. Materials and Methods

### 2.1. Participants

A total of 241 team sports players (handball and basketball) belonging to different clubs in the Murcia region and Valencia communities participated (160 boys, 66.4%; 81 girls, 33.6%), with an average age of 15.36 years (SD = 1.07). According to age, 60.6% (*n* = 146) of the participants were <15 years, while 39.4% (*n* = 95) were >15 years old. Most of them stated that they had an average of 1.56 years of experience as a federated player in their sport (SD = 0.50) and they spent 1.93 training sessions per week (SD = 0.36).

### 2.2. Measurement Instruments

Resilience scale. The Spanish version of the Resilience Scale (RS-14) [43] was used, along with the 14-Item Resilience Scale (ER-14) [44], which is an abbreviated version of the original 25-item version [45]. This scale measures a person’s degree of individual resilience when such is considered a positive personality characteristic, one that allows people to adapt to adverse situations. The scale is composed of 14 items grouped in two dimensions that measure personal competence (11 items: self-confidence, independence, decision, ingenuity and perseverance, etc.; e.g., “I am not afraid to suffer difficulties because I have already experienced them in the past”), and the acceptance of oneself and life (3 items: adaptability, balance, and flexibility and stable life perspective; e.g., “I am a person with adequate self-esteem”). Every item on the scale should correspond with the respondent’s feelings. The answers were on a 7-point Likert-type scale that ranged from strongly disagree (1) to strongly agree (7). The internal consistency for the sample collected was satisfactory (α = 0.80).

Competitive State Anxiety Inventory-2R. The Competitive State Anxiety Inventory-2R (CSAI-2R) [46] comes from the original CSAI-2 version consisting of 27 items [20]. The Spanish version of this second revision, which was validated for athletes, was used in this study [47]. The CSAI-2R is a specific inventory for the sports context, but with a one-dimensional conception of the state of anxiety. The scale is composed of 16 items grouped in three dimensions that measure somatic state anxiety (6 items; e.g., “My heart is racing”), self-confidence (5 items; e.g., “I am confident”), and cognitive state anxiety (5 items; e.g., “I worry that others will be disappointed with my performance”). Each item of the scale had to be answered in terms of how the player felt just before the competition. The answers were collected on a 4-point Likert-type scale ranging from none (1) to very much (4). The internal consistency for the collected sample was satisfactory (α = 0.78).

### 2.3. Procedure

The study was carried out in different clubs and sport federations of Murcia region and Valencia community (Spain). A letter explaining the objectives of the research and how it was to be carried out, accompanied by two models of informed consent and permission (one for the parents and another for the young athletes), together with a copy of the measures, was sent to the clubs and federations (first for presidents) before the data collection. The questionnaire was administered by the researchers in the sports facilities of the clubs during the training sessions and completed by the participants in 20–30 min. All the participants were informed of the objectives and of their rights as participants in the study, as well as of the voluntary nature and the absolute confidentiality of the answers and handling of the data. It was explained that there were no correct or incorrect answers, so that participants would answer with the most sincerity and honesty. This study was carried out in accordance with the ethical guidelines of the American Psychological Association (APA). The protocol was approved by the Ethics Committee of the Universidad de Murcia (ID: 1494/2017). All subjects gave written informed consent in accordance with the Declaration of Helsinki [48].

### 2.4. Statistical Analysis

Through the analysis carried out with the statistical software SPSS. 23.0, descriptive measures and homogeneity (X^2^ Chi-squared) were obtained for the distribution of the observed sample, internal consistency of the instruments (Cronbach’s alpha), differential analysis (*t*-test, multivariate analysis and effect size with etha-square) to describe any differences in the sample (according to gender and sport experience), correlations, and multiple regressions (5000 bootstrap samples with bias-corrected confidence intervals 95.00) for the determination of the strength of the relationship between the study variables.

## 3. Results

### 3.1. Descriptive and Differential Analysis

The sample distribution reflected the existence of a statistically significant association between both variables (Table 1) for the achievement of normality, which was assumed to be a link of dependence between the distributions by gender and sport experience (years). Similarly, this circumstance was again obtained when considering the distribution by gender and the hours of weekly training.

As can be seen in Table 2, the sample reflected higher means of self and life acceptance in terms of resilience, while higher means of self-confidence were observed in the perception of anxiety. Participants’ response tendency (asymmetry) tended to be concentrated between 5 and 7 points (Likert response orientation was 1–7) for personal competence as well as for acceptance, with pronounced peaks at 5 and 8 (kurtosis). Likewise, the participants’ response tendency in anxiety perception tended to concentrate (with a Likert response orientation from 1 to 4), between 2 and 3 points for somatic anxiety, and between 2.5 and 3.5 for cognitive anxiety and self-confidence, with pronounced peaks at 2.5 for somatic anxiety and 3 for cognitive anxiety and self-confidence.

Differential analysis (Table 3) showed the existence of statistically significant differences with respect to gender and sports experience. By gender, resources for acceptance (*p* < 0.02) were shown in favor of girls and self-confidence (*p* = 0.00) in favor of boys. The effect size of the established comparisons indicated that all the variables are very high, except for competition and cognitive anxiety, aspects that were repeated in the analyses carried out both by gender and by years of sports experience. According to sports experience, the most experienced young athletes showed differences in acceptance (*p* < 0.03) and self-confidence (*p* < 0.01).

In order to check whether there were differences in resiliency resources and anxiety responses based on gender and years of sports experience, an analysis of multivariate variance (MANOVA) was carried out with the scores obtained in all the variables under study (Table 4). The results (Pillai’s Trace) indicated statistically significant differences in favor of girls (F_(5.232)_ = 9.08; *p* < 0.01) with the highest magnitude of the effect (η^2^ = 731; *r* = 0.46). Statistically significant differences were shown based on years of sports experience in favor of the younger, most experienced ones, but the interaction with the gender variables was significant (F_(5.232)_ = 7.512; *p* > 0.03), with a moderate effect size (η^2^ = 779; *r* = 0.51). More specifically, analysis of the variance of effects between subjects showed significant differences in competence, acceptance, cognitive anxiety, and self-confidence by gender (in competence and self-confidence in favor of boys, and in cognitive anxiety and acceptance in favor of girls). In the years of sports experience, the differences were in acceptance, cognitive anxiety, and self-confidence in favor of the younger, more experienced ones. No significant differences were shown in the gender*sport experience interaction.

### 3.2. Correlational Analysis

Statistically significant two-way relationships appeared between the variables under study (Table 5), so that older athletes had higher perceptions of competence as shown in their lower levels of managing resiliently. Acceptance was inversely and significantly related to both cognitive and somatic anxiety. Conversely it was directly related to self-confidence and logically to a high perception of competence. All this led us to think that higher perceptions of resilient acceptance had a direct influence on the athlete’s ability to interpret anxiety. The relationship between the two resilient variables (acceptance and competence) with the development of self-confidence in a young handball player is very significant, in a positive way.

### 3.3. Multiple Regression Analysis

The results of the regression analysis (Table 6) indicated a model in which as the athletes showed better levels of acceptance, competence, and cognitive anxiety, they also reduced their somatic anxiety indicators, and increased in self-confidence, where F_2.239_ = 23.59; *p* < 0.01 explains 33.5% of the total variance.

The results of the regression analysis (Table 7) also indicate a model in which younger athletes had better levels of acceptance and self-confidence and more competence, where F_2.239_ = 33.96; *p* < 0.01 explains 42.1% of the total variance. On the other hand, acceptance was achieved through the predictive model established by an increase in competence and self-confidence, and the existence of low levels of cognitive anxiety.

## 4. Discussion

The objectives of this study were as follows: (a) to describe the levels of resilience and competitive anxiety, (b) to analyze the differences in terms of gender and years of sports experience, (c) to study the relationship between resiliency resources and competitive anxiety, and (d) to examine the variables that predict young players’ resiliency resources and self-confidence.

### 4.1. Resilience Levels and Competitive Anxiety

The results indicated that both the levels of self-acceptance and life and competence were high. These outcomes imply that athletes best face adversity with high levels of resilience [21]. This finding is also consistent with previous studies, most of which were conducted in team sports such as handball and football with young sport team athletes [28,30,31,32,35]. As for anxiety levels, the results reflected moderate and high levels of self-confidence. It should be noted that moderate levels of anxiety could optimize athletes’ performance [49]. These results are consistent with those found in other studies conducted with samples belonging mostly to individual sports [12,16,18,50] and contradict the high levels of somatic [14,17] and cognitive [19] anxiety reached by others.

### 4.2. Differences in Levels of Resilience and Competitive Anxiety Depending on Gender and Years of Sports Experience

In relation to resilience, the results showed that acceptance levels were higher in boys, while competence levels were higher in girls. The literature indicates that it is usually boys who show higher levels of resilience due to an increase in physical self-concept and that girls are much more affected by the sports context [51]. In terms of sports experience, it was shown that both acceptance and competence levels were higher in players with greater sports experience. These results are consistent with findings in previous studies in similar samples, most of which were conducted with team sports [31,32].

On the other hand, higher levels of somatic anxiety were found in girls and cognitive anxiety in boys. The literature provides different explanations for why girls tend to have higher levels of anxiety than boys. Abrahamsen et al. [12] attributed the difference to boys’ interpretation of performance; Brunet and Sabiston [18] attributed it to a greater perception of competence and motivation in boys; Peñaloza et al. [50], León-Prados, Fuentes and Calvo [51], and Arbinaga and Caracuel [52] attributed it to girls’ greater concern about the loss of social recognition caused by poor performance. In regard to self-confidence and gender, studies were found that differed from the results reported. Morillo et al. [19] and Peñaloza et al. [50] revealed higher levels of self-confidence in boys. The controversy between the results of the study and those found in the literature may be due to the age difference of the analyzed sample. Finally, in other studies such as the one conducted by Montero, Moreno-Murcia, González, Pulido, and Cervelló [53] there were no statistically significant differences in relation to gender, nor in the levels of anxiety or self-confidence.

As regards the relationship between anxiety levels and sports experience, although no statistically significant results were found, higher levels of somatic anxiety and cognitive anxiety and lower levels of self-confidence were reported in athletes with less sports experience, thus coinciding with findings from previous studies conducted with samples belonging to both team and individual sports. Hanton et al. [14] with adults and Hernández et al. [16] with youths showed that athletes with greater sports experience faced anxiety with higher levels of self-confidence and managed to interpret anxiety better than those with less experience.

### 4.3. Relationship between Resiliency Resources and Competitive Anxiety

The results of the correlational analysis reflected a negative association between the two dimensions of anxiety (cognitive and somatic anxiety) and of acceptance, showing that competence was negatively related to somatic anxiety and positively to cognitive anxiety. The papers reviewed showed that the two dimensions of resilience were negatively related to those of anxiety [37,38,54,55]. Similar studies were conducted in team sports such as handball, basketball, football, and rugby, and found that youth athletes with higher levels of resilience managed to moderate their anxiety levels significantly, recovering or adapting better to threatening situations [38,41,42,54]. In addition, resilient athletes may be better able to protect themselves in the face of adversity, while those with lower levels may have greater failures, thus causing low self-confidence and higher levels of anxiety [41].

### 4.4. Resiliency Resources and Self-Confidence Predicting Variables

The regression analysis showed that self-confidence is likely to increase as athletes have better levels of acceptance, competence, and cognitive anxiety and that it is likely to reduce their indicators of somatic anxiety. These results are in line with the findings of Ortin-Montero et al. [42] in their study of handball players, in which the players with higher levels of resilience showed higher levels of self-confidence and coped better with adverse situations.

Furthermore, the regression analysis also indicated that younger athletes have higher levels of competence with higher levels of acceptance and self-confidence. In addition, the acceptance was greater when combined with competence, self-confidence, and less cognitive anxiety. The literature reviewed shows that young athletes tend to be the most resilient [32,55].

The present study, in addition to providing new information on the relationship between resiliency resources and anxiety levels in athletes, has some limitations that should be highlighted. One of them is that the generalization of the results is limited, due to the sample (cadets and juniors), so these results should be considered as preliminary, needing future replication in other categories such as children and other sports, thus covering all ages of adolescence and sports, and both team and individual sports.

In terms of strengths, it is important to highlight the novel information provided in relation to the field of study and the instruments used, as these have been used in a multitude of works and with a high degree of consistency. Likewise, it would be necessary to complement future studies with the analysis of other variables related to anxiety and resilience such as stress and optimism. Finally, another interesting aspect would be to be able to contrast resiliency resources and anxiety levels in collective and individual sports and see the possible differences that are generated.

## 5. Conclusions

The consequent negative relationship between resilience resources (especially acceptance and competition) and levels of competitive anxiety, is a reflection that to enhance the former makes it possible to regulate up to control the latter. Despite the existence of indicators of cognitive anxiety (e.g., recurrent thoughts or rhyming), coaches and athletes need to understand that they are also indicators of a necessary activation for psychological functioning. Channeling such a process through psychological training of different skills will enhance the capacities for self-confidence. Making young athletes grow up on self-acceptance (e.g., reducing the belief that they cannot lose), for the younger ones, and on the perception of competition (e.g., learning to observe their improvements) already in adolescence, will undoubtedly consolidate key aspects for an effective and adapted psychological response to sports situations.

Highlighting how to work on skills, such as understanding situations of uncertainty, cooperation, or the search for social support at early ages and with strategies that integrate such resources in the dynamics of training and sports growth, will enhance the resources for the much needed coping with stress and anxiety, as well as consolidate beliefs and attitudes that can reduce dysfunctional responses that alter (e.g., blaming others or oneself, creating fears of failure after several mistakes).

## Figures and Tables

**Table 1:** Distribution and homogeneity of intervariable variances (gender, sport experience, and weekly training hours).

Chi-squared (*X^2^*)_2_ = 19.26; *p* = 0.402 *	**Sport Experience**			
	**≤** **5 years (*n* = 106)**		**> 5 years (*n* = 135)**	
	**Mean (SD) age**	***n* (%)**	**Mean (SD) age**	***n* (%)**
	15.67 (0.51)		15.14 (0.49)	
Boys = 160 (66.39%)	Boys *n* (%)	78 (73.58%)		82 (60.74%)
Girls = 81 (33.61%)	Girls *n* (%)	28 (26.42%)		53 (39.26%)
Chi-squared (*X^2^*)_2_ = 14.39; *p* = 0.236 *	**Weekly Training Hours**			
	**≤** **2 h/week (*n* = 17)**		**> 2 h/week (*n* = 224)**	
	**Mean (SD) age**	***n* (%)**	**Mean (SD) age**	***n* (%)**
	15.71 (1.05)		15.33 (1.07)	
	Boys *n* (%)	17 (100%)		143 (63.84)
	Girls *n* (%)	0		81 (36.16%)

* *p* < 0.05.

**Table 2:** Means, standard deviations, and measures of dispersion of the variables studied for the sample.

*n* = 241	Means	Standard Deviation	Asymmetry	Kurtosis
Competence	5.46	0.79	−0.47	0.29
Acceptance	7.37	1.56	−0.20	0.10
Somatic anxiety	2.45	0.77	0.18	−0.85
Self-confidence	3.01	0.59	−0.34	−0.05
Cognitive Anxiety	2.75	0.71	−0.29	−0.37

**Table 3:** Differential analysis (*t*-test), according to the gender and sports experience of the participants.

*n* = 241; gl_2.239_	(*n* = 160) Girls	(*n* = 81) Boys	*t(p)*	*d*	(*n* = 106) ≤5 years	(*n* = 135) >5 years	*t(p)*	*d*
	M (SD)	M (SD)			M (SD)	M (SD)		
Competence	5.39 (0.79)	5.58 (0.86)	−3.43	0.40	5.45 (0.81)	5.46 (0.78)	1.03	0.03
Acceptance	7.83 (0.78)	7.05 (0.86)	2.25 (0.02 *)	−0.64	7.33 (0.70)	7.90 (0.64)	−1.62 (0.03 *)	0.07
Somatic anxiety	3.21 (0.73)	3.14 (0.76)	−3.21	0.98	2.48 (0.75)	2.43 (0.78)	1.08	−0.11
Cognitive anxiety	3.04 (0.58)	2.94 (0.62)	−4.13	−0.28	3.03 (0.66)	3.00 (0.54)	1.48	−0.07
Self-confidence	2.61 (0.68)	3.02 (0.68)	−4.32 (0.00 **)	1.01	2.68 (0.73)	3.40 (0.69)	−1.83 (0.01 *)	0.78

* p < 0.05; ** *p* < 0.01

**Table 4:** Analysis of multivariate variance of resiliency resources and anxiety, according to gender and years of federated sport experience.

	≤5 Years		>5 Years		F_(5.232)_ Gender	F_(5.232)_ Years	F_(5.232)_ Gender × Years
	Boys (*n* = 78)	Girls (*n* = 28)	Boys (*n* = 82)	Girls (*n* = 53)			
	M (SD)	M (SD)	M (SD)	M (SD)			
Competence	5.85 (0.79)	5.18 (0.83)	5.72 (0.78)	5.33 (0.77)	0.05 *		
Acceptance	6.51 (0.59)	7.26 (0.88)	7.32 (0.38)	7.68 (0.52)	0.01 *	0.03 *	
Somatic anxiety	2.41 (0.73)	2.64 (0.75)	2.23 (0.73)	2.75 (0.77)			
Cognitive anxiety	2.61 (0.73)	2.94 (0.65)	2.93 (0.64)	3.26 (0.70)	0.02 **	0.02 *	
Self-confidence	3.10 (0.62)	2.82 (0.72)	3.53 (0.53)	3.21 (0.56)	0.00 **	0.00 **	

* *p* < 0.05; ** *p* < 0.01

**Table 5:** Correlational analysis between resilience dimensions and anxiety responses.

	M	1	2	3	4	5	6
1. Somatic anxiety	2.45 (0.77)	1	0.533 **	−0.123	−0.073	−0.193 **	−0.110
2. Cognitive anxiety	3.01 (0.59)		1	0.049	0.013	−0.144 *	−0.125
3. Self-confidence	2.75 (0.71)			1	0.547 **	0.387 **	−0.033
4. Competence	5.46 (0.79)				1	0.494 **	−0.164 *
5. Acceptance	7.37 (1.59)					1	0.051
6. Age	15.36 (1.07)						1

* *p* < 0.05; ** *p* < 0.01

**Table 6:** Variable predictors of young players’ self-confidence.

Dependent Variable	Step	Independent Variables	Beta	*t*	*p*
Self-confidence					
*R* = 0.579; *R^2^* = 0.335; *F*_2.239_(*p*) = 23.59(0.00)	1	Age	0.023	0.34	0.46
	2	Competence	0.354	7.46	0.00 **
	3	Acceptance	0.057	2.35	0.01 *
	4	Somatic anxiety	−0.100	−2.02	0.04 *
	5	Cognitive anxiety	0.112	2.09	0.03 *

* *p* < 0.05; ** *p* < 0.01

**Table 7:** Predicting variables of resiliency resources in players.

**Dependent Variable**	**Step**	**Independent Variables**	**Beta**	***t***	***p***
**Competence**					
*R* = 0.648; *R*^2^ = 0.421; *F*_2.239_(*p*) = 33.96 (0.00)	1	Age	−0.123	−3.30	0.63
	2	Acceptance	0.177	6.31	0.00 **
	3	Somatic anxiety	0.021	0.34	0.73
	4	Cognitive anxiety	0.018	0.26	0.79
	5	Self-confidence	0.543	7.46	0.00 **
**Dependent Variable**	**Step**	**Independent Variables**	**Beta**	***t***	***p***
**Competence**					
*R* = 0.648; *R*^2^ = 0.421; *F*_2.239_(*p*) = 33.96 (0.00)	1	Age	−0.150	1.836	0.68
	2	Acceptance	0.823	6.308	0.00 **
	3	Somatic anxiety	−0.154	−1.159	0.24
	4	Cognitive anxiety	−0.217	−1.515	0.02 *
	5	Self-confidence	0.407	2.358	0.06 *

* *p* < 0.05; ** *p* < 0.01
